# Cremation

**Published:** 1881-08

**Authors:** C. W. Purdy

**Affiliations:** Chicago


### ARTICLE VII.

CREMATION. By Dr. C. W. PURDY, of Chicago.

Death made its advent into our world through the agency of a dreadful crime, and ever since Cain thought to conceal the body of his victim with earth (the first burial) has the disposal of the dead been an anxious question with the living.

Many contradictory and superstitious customs of disposing of the dead were in vogue among ancient nations and barbarous tribes, but these are of little use to us as precedents. Herodotus, Cicero and Lucian inform us that some Asiatic nations feasted on the slain, and murdered the sick and aged; that others threw their dead to ferocious animals; that others cast them into seas, lakes and rivers; that the Scythians buried them in the snow. According to Spondanus, the "Syrcanians flung their dead to the dogs; some Indians left them to vultures; the Celts took from them the vertex of the cranium, which they set in gold for goblets." But as man became more civilized, the voice of religion, reason and policy called for the careful interment of the dead.

The Egyptians attached to public burial an idea of the greatest honor, as indeed did also the Greeks and Romans, and it was considered the highest recompense of virtue, and hence the desire of becoming worthy of funeral obsequies was universally cherished. It was a crime to disturb the repose of the dead in their last abode. A reverence for tombs thus became a part of religious worship, and to pay the last duties to the dead was held a sacred obligation.

Man, impelled by his passions, always oversteps the bounds of reason. The affectionate zeal of afflicted relatives invented at length the art of giving a kind of perpetuity to the inanimate form, and thus the early Egyptians embalmed the dead; using every art capable of preventing the action of the air upon the body to prevent corruption, and to secure it in such a manner, that it might be kept without danger to the living.

Self love gave much encouragement to this art. It was believed that as long as the body remained entire, the soul hovered near it. Thus they were kept as a most precious family deposit, and a sacred civil pledge. The inevitable result followed of delaying Nature's processes. Contagious diseases broke out in the midst of the Egyptians and baffled every remedy ; and all the embalmed were obliged to be removed to a distance. The great number of dead, after the carnage of battles, obliged them to resort to burning. In time the old practice was completely changed; and tombs and vases were only used for the ashes of the funeral pile.

Long wars, frequent transmigrations, ruin and rebuilding of cities in time overturned whole countries, and bones confided to dust for centuries were exposed and disturbed unavoidably. As this was a profanation nothing short of the most awful sacrilege, it resulted at length in the general determination to adopt burning of the dead. They even excluded the venerated ashes from within the walls of cities; and deposited the urns in places consecrated to burial. The highways were for a long time bordered with tombs and slabs of marble covered with inscriptions.

"One consequence of stationing tombs upon the roads beyond the gates was that towns attacked were not as liable to slaughter and destruction; the citizens would leave their walls for the defense of those sacred remains."

We will find the elements of our own customs by a glance at the rites practiced by the Hebrews, Greeks and Romans.

Inhumation was practiced perhaps more universally by the Hebrew than any other race, and hence the origin of the Christian burial; and yet this is the only Jewish custom retained by them. Abraham bought from the children of Heth the cave of Hebron and deposited there the corpse of Sarah. He himself was also buried there, as was Isaac, Rebekah and Leah. It is believed, as commentators declare, that the remains of all the illustrious patriarchs were assembled in the cave of Hebron with the bones of Abraham. Vaults dug in the hill of Zion, under the foundations of the temple, and in the royal gardens, were destined for the last abodes of the kings of Judah.

The course of time brought but slight changes in the practices of this people, although they underwent such eventful vicissitudes.

Although burial was the chief method of disposing of the dead, it was not the exclusive one. Among the great, especially kings, some secured the honor of embalming, and cremation was occasionally adopted for the same purpose (Jer. xxxiv. 5); probably this custom was of short duration and only peculiar to a few.

The bodies of Saul and Jonathan, however, were burnt to ashes by the people of Jabesh-Gilead in order to rescue them from the insults of the Philistines. We are also told that "a continual fire that consumed the carcasses and the filth of the city, burned always in the deep pit of Topheth in the valley of the children of Hinnom. (Isaiah xxx. 33.)

To the Greeks belong the credit of more uniform practice of burning the dead than any other nation of ancient times. They inclosed the urns which contained the ashes of their dead in private houses, within cities, and sometimes in their temples. Some traced back the origin of the custom to Hercules, who wished to carry to King Licinius the last remains of his son Argivus who perished in battle.

In the history of the Roman nation, nothing is perhaps more interesting than the endless variety of laws, rites and forms which they observed toward the dead. One half of the ruins or emblematical antiquities remaining, relates to this.

Numa Pompillius was buried on the Janiculine Hill; the succeeding kings were buried in the Campus Martius, a large plain along the Tiber. Ancient Rome permitted none of her citizens to be buried within her walls, save the vestal virgins. In course of later times, however, great heroes were allowed the same honor.

"During the many wars of the Republic and the incursions of the Barbarians, the impossibility of interring all human remains left exposed, necessitated their disposal by burning. Again, experience had taught them that they could not with safety bury their dead among themselves and their dwellings. To protect the remains of their venerated chiefs or companions in arms, the funeral pyre was adopted in imitation of the Greeks. The ashes were carefully collected by the friends of the deceased, enclosed in an urn and deposited in a monument, or in the catacombs, some of which still exist." (Pascalis.)

Thus we observe that three principal methods of disposing of the dead have been practiced: viz., inhumation, embalming, and incremation. The first originated with the first family of man, and has been more or less in practice to the present time. The second, embalming, if not originating with, at least was most extensively practiced by the Egyptians, and the perfection of this art attained by them has long since been lost to themselves and to the world.

Cremation of the dead was in more or less general practice among all the ancient nations of the world, but more largely so by the Greeks and Romans. Of the origin of this practice we are not reliably informed by historians. It is more than probable that it arose through a desire to preserve the dead from the passions and fury of the living, in those ages when man's barbarous passions impelled him to pursue his victims after death. Witness the fierce Achilles tying the body of Hector by the heels to his chariot, and driving around the city of Troy.

But the general adoption of cremation by the Egyptians, Greeks and Romans, was always the result of necessity for the protection of the living. About the end of the fourth Christian century its practice was discontinued, owing to the growing power of Christianity, whose doctrine of the resurrection, with its strong attachment to inhumation, brought a great influence to bear against it.

One of the most solid arguments in favor of cremation is from a sanitary standpoint; and this is what interests us more especially as medical men.

From medical societies should spring the germs of sanitary excellence, and it is eminently proper therefore, that we should give careful consideration to matters looking to so important a sanitary reform.

To appreciate more fully the sanitary advantages of cremation, let us first look at the ghastly record of our present burial system.

"The history of vaults, grave-yards and cemeteries is one long tale of desecration, robbery and outrage; coffins have been rifled from time immemorial for treasures; there is scarcely a museum in the world which does not bear ample witness of this practice of spoliation; even professional grave-diggers have dug up the bodies of the high born for the purpose of finding rings and other treasures buried with them."

The London *Quarterly Review* (No. 42, p. 380), says "that many tons of human bones every year are sent from London to the north, where they are crushed in mills and used as manure."

It is impossible to preserve the dead by burial from the outrages of the living. Grave-diggers are often employed to remove bones; as a rule they are not scrupulous and are often drunk. "Where are the thousands who were buried in the heart of Paris, and slept there for centuries in the tombs of the Innocents? You may to-day gaze on their bones and comment over their skulls. By order of the Minister of Police they were all dug up in 1707 and carried off to the catacombs. The bones were cleaned and arranged in the galleries; in one the arms, legs and thighs, intersected by rows of skulls; the small bones thrown in heaps behind. 'They were bourn there, we are told, by priests and tapirs!'" (Hawes.)

A New York correspondent affirms "that a number of men were recently employed as grave-diggers in one of the old grave-yards of that city. Bodies were offered for sale on the ground to a party of medical students, who shrank from the horrors they witnessed. One coffin was found to contain a heavy decomposed mass like spermaceti; it was used to grease the axle-tree of a cart. Another coffin contained the body of a woman aged twenty, as the inscription announced. She had rested for 100 years—laid there with what tears, what tender regrets of husband, brother or mother! but now her head was rudely seized and kicked like a foot-ball from one ruffian to another."

Previous to the passage of the Burial Acts, 1852–56, violation of graves in Scotland, England and Ireland was systematic. "In most crowded burial grounds there was a bone-house; and, in many, a burning ground for coffin wood."

You paid for your grave; your friends thought you would lie undisturbed. If you were in a vault, your coffin might still be there; but after a certain number of years not your bones. Your bones would be in the bone-house, or sold for manure; your coffin burnt. In many parts of London coffin-wood was used habitually for fire-wood, and coffin furniture (second-hand plates, nails, etc.,) was a well-known article with marine store dealers." (See Walker, "Gatherings from Grave-yards.")

A surgeon visited the burial-ground in Portugal street, April 27, 1839. He found two graves open, several bones lying about; and heaps of coffin wood lying about, some quite fresh, awaiting removal for firewood.

A gentleman writes to the London *Times*, June 25, 1838, of this same grave-yard: "I was shocked to see two men employed in carrying baskets of human bones to the back of the ground, through a small gate. I have twelve of my nearest relatives consigned to the grave in that ground."

A writer in the *Weekly Dispatch*, Sept. 30, 1838, says of St. Giles church-yard: "It is full of coffins up to the surface. Coffins are broken up before they are decayed and bodies removed to the bone house before they are sufficiently decayed to make their removal decent. This bone house is a large round pit; into this had been slid from a wheel-barrow, the but partly decayed inmates of the smashed coffins. On the north side was a man digging a grave. He was quite drunk."

This over-crowding of cemeteries and burial-grounds is one of the most dangerous abuses to which the system of burial is subject, and this is bound to occur in every grave-yard situated in populous districts. It only remains a question of time.

St. Giles and St. Pancras, of which we have been speaking, are said to be literally saturated with corpses; yet long after such was the case, the hearses streamed in to deposit their ghastly freight of human merchandise.

Look at most large towns—once a rural village, to-day a crowded city with a pest-house in its center. Such in time is the history of most burying-grounds. Look at those hundreds of plague acres closed by Acts of Parliament in England, and think what overcrowding did there.

Nor need we go so far from home for examples in this matter; look at the overcrowded condition of the cemetery of Trinity Church, New York, and on the malignant fever induced by overcrowding there in 1822, for an account of which see *Commercial Advertiser*, Aug. 7, 1822. Part of this same cemetery is now walled up with its contained bodies, and the traffic of Broadway resounds above the once consecrated ground.

It is only a question of time when our own beautiful Graceland, Calvary and Rose Hill will have been overcrowded, and perhaps swept away by the resistless march of our city's marvelous and unprecedented growth. Already the voice of mirth and the prattle of our children may be heard any day over the sod of what, only thirty years ago, was our principal cemetery. Only a few years ago, and each day the dark caravans might be seen wending their solemn way northward to deposit the dead. In the same spot now is our most frequented and popular of public parks.

Where are the bones of those laid to rest there by our early settlers, and the founders of our city's greatness ? Some have been carted away for a short time farther north, while some—many indeed—still lie beneath those public walks and driveways, while the wheels of revelry, and the tread of pleasure seekers sounds constantly over their remains.

" The church-yard sentiment is a false one, because it is untrue to facts. You think of the body in its quiet, hermetically sealed tomb; but time knows no such seal. The protecting slab, the solemn walls about the consecrated ground, are all changed tomorrow. The church-yard sown with flowers, its groves of drooping willows, the quiet, peaceful dead,—it is all a fancy. The voice of history tells us that in the past there has been no such seclusion, and what is to change the course of events in the future. The world is the world of the living, and attempts to make it a dead-house will break down in the future as it has in the past."

From the New York *Commercial Advertiser*, August 17, 1822, see the following: " It is not long indeed since we have seen, in this populous city, the mangled remains of the dead transported from one burying-ground to another. We have seen two church-yards opened, and deeply broken over an extensive surface, leaving exposed to our reluctant curiosity shattered limbs of corpses, and the decayed coffins, until they were closed by a range of brick vaults.

"These extend now a great way into the streets, literally under the pressure of a thousand horses, carts and carriages. An earthquake might in an instant throw up from under our feet the stings of death, the poison of human putrefaction, and create unexpectedly horrid scenes and cruel contrasts. It is well known that our large cities have, within a few years, extended much beyond their former suburbs; it has been necessary to build in many instances on what was formerly a burying-ground,—to expel, as it were, the dead for the accommodation of the living."

The over-crowding of grave-yards has led to a system of what grave-diggers call "management"; this is, simply making room for more bodies, and in some of the cemeteries has been carried to a disgraceful extent.

"The vaults of Enon Chapel and Clement's Lane are 59 ft. 3 in. by 28 ft. 8 in., allowing an average of 9 ft. per person. 200 would quite fill the whole area; fill that area six times, then the coffins lie in tiers six deep, and the whole space could not possibly contain more than 1200, and yet instead of 1200, between 10,000 and 12,000 were crowded into those vaults at various times! What became of them? They could not all rest there, thousands of them had to be managed." (Hawes, p. 27 & 28.)

The burial-ground of St. Cuthbert is situated in the center of the city of Edinburgh. (We quote from the *Scotsman*, March, 1874.) In the last fifteen years, 10,800 bodies have been placed there. Graves were re-opened up to 1874, some every seven years, some every three years; coffins with no plates were usually broken open, the remains were often not ripe (decayed), the coffin wood was burned, the remains heaped back pell mell upon others, to make room for more. At last but one or two inches of earth could be placed between the coffins. When all this came out in court the sheriff's address amounted to a defense of St. Cuthbert's."

The following is quoted as a notorious fact in the current prospectus of the Woking Necropolis Co. of London, 1874:

"In 1850 the Board of Health condemned the cemeteries of Highgate, Kensal Green, Norwood, Nunhead, and Brompton, and declared that they must be closed in the interests of public health. A central burying-ground was to be purchased at Abbey Wood, and Parliament was to buy up the old cemeteries. It bought

Brompton only, and closed it—no, used it, and is using it now. The government is accordingly using at high pressure and remunerative rates, a grave-yard condemned by its own board over a quarter of a century ago."

The necessary limits of this paper will not permit our dwelling at greater length on this over-crowding system of burying the dead. Those who wish to investigate the extent to which it has been practiced, and the sickening results, we would refer to a work published by Mr. Walker, entitled "Gatherings from Graveyards."

Mankind, until late years, have not devoted much consideration to the investigation of causes which might so far corrupt the atmosphere, as to create mortal diseases during marked periods of time; or why these plagues should appear in the vicinity of stagnant pools, shallow waters, receptacles of dead animals, or vegetable substances, in the neighborhood of slain after battle, in cities ravaged by famine; why columns of air have been rendered so noxious that the very birds that attempted to fly through them fell and perished; why the winds disseminating these aërial poisons, transported the breath of pestilence into rich, fertile, and thickly inhabited districts.

With the story of such terrible devastations the pages of history are filled. Putrid and malignant fevers, and periodical diseases often make their appearance in populous cities, without any apparent cause. May not this cause, known to us principally by its fatal results, be the practice of interment in the very midst of our dwellings? Epidemics which have laid waste whole cities have originated in the practice of burying in temples and churches. Diodorus, of Sicily, speaks of pestilences that were produced by the putrefaction of animal substances. Egypt is ravaged almost every year by malignant fevers, and from that country small-pox has spread over all the earth. The waters of the Nile leave with their slime an immense number of aquatic insects, which, as they corrupt, fill the air with pestilential miasmata. Lucan speaks of the ravages of an epidemic in the army of Pompey, near Durazzo; this was caused by the carcasses of horses, killed and left upon the field. Ammienus Marcellius informs us that the camp of Constantine the Great, was desolated in consequence of the same imprudence. Frequently the corpses scattered over the field after battle have been the source and origin of disease and death on a large scale. The war of the Swedes in the seventeenth century, occasioned in like manner the great pestilence of Poland. Long and obstinate wars had the same effect in Austria, Spain, and in many other countries.

As we write at present, a plague is devastating Nedjeff, in Mesopotamia. "In this place is the grave of Ali, son-in-law of the prophet Mahomet. To this place his followers send the dead bodies of their friends and relatives, because they believe to be buried near this spot will assure their souls admission to Paradise. Caravan after caravan arrive there daily and deposit their ghastly freight for interment. The whole country about Nedjeff has become one vast grave-yard. The frequent floods occurring in the Euphrates, inundate all the land on both sides of the river. The light covering of earth is swept from the coffins, they fall to pieces, and thousands upon thousands of corpses are left rotting under the oriental sun, filling the air with their pestilential odors." Is it any wonder that a correspondent writes, May 2, 1881, that during the week just ended an average of 56 persons died daily out of a population of 6,000.

Both animal and vegetable substances, if subjected to heat and moisture at the temperature of 72° F. are converted in a little time into a variety of gases, carbonous, carbonic acid, carburretted, sulphurretted, phosphoretted, hydrogenous, etc., with azote, all of which are deleterious and deadly in their effects when inhaled into the system. It follows that carcasses can not be long exposed to heated air without becoming hurtful to persons in the vicinity of them. It has been argued, that provided a thick or heavy layer of earth be interposed between the dead material and the external air, no such gases can reach us to do any injury, and that they cannot remain long in their specific nature, but must soon be absorbed or decomposed and destroyed. This argument is not in keeping with the laws of philosophy, or practical facts. Layers of earth, even to seven feet in depth, can no more intercept the transmission of gas into the atmosphere than they can preclude the filtration of water. The power of the one is to descend and the other to ascend through a permeable medium. Water descends by its weight and affinity or cohesion. Gas ascends by a double power ; first, by the impulse given to each column of it in the focus of fermentation, from which it is evolved with such a force and elasticity that it requires a thick covering of lead and wood to check it in vaults ; secondly, by atmospheric pressure, the action of which upon the surface of a grave 32 square feet, is equal to a weight of about 71,424 lbs. The only advantage in the depth of a grave is that of rendering septic formation more slow, on account of the slow diffusion of heat through the deep ground ; the want of this is replaced in proportion to plentiful rains. We do not deny the gravity of gases under consideration, and that they do not rise in the air to a great height; but this is no protection against them : winds will not only raise but transport them to our dwellings. When such air, loaded with putrid emanations, remains stagnant, where it is little renewed, its effects become most deadly. Experience has over and over again shown that diseases of the worst kind, such as malignant, putrid, and exanthematous fevers are the fatal consequences. (See Dr. Felix Pascalis on Intra-mural burials.

"On the 17th of August, 1744, a body was conveyed to a vault in the Parish of Notre Dame, of Montpellier, attended by a numerous procession. While lowering the corpse, a man first went down to support the coffin, and fell senseless ; another followed to assist him, and though drawn out in time, nearly perished; the third had the courage to descend with a rope around his waist, and had he not been drawn up immediately would inevitably have died; the fourth, a strong and vigorous man, trusting to a robust constitution, dared the danger, and died as soon as he had entered the vault; the fifth came out once to recover strength and returned the second time, staggered from the ladder and fell dead.

"Dr. Haguenot was commissioned to investigate the matter, and having had the vault re-opened, made the following experiments :

"1st. The cadaverous fetor was so tenacious as to adhere a long time to any substance which was left a few moments in the vault.

"2nd. Lighted tapers, chips, paper and tarred ropes, when brought to the edge of the vault, were instantly extinguished, as completely as if dipped in water.

"3d. Small animals, dogs and cats, became instantly convulsed and died in a few minutes, birds only lived a few seconds on breathing the impure atmosphere.

"4th. The vapor or gas was caught in glass vessels, and, after being kept for six weeks, afforded the same qualities and produced the same results."

The whole of these were witnessed and certified by Dr. Haguenot and a committee of the faculty of the University of Montpellier.

G. A. Walker, author of the book previously referred to, declares that "of all the grave-diggers he has spoken to, not one has wholly escaped the effects of poisoning; many had been overpowered by the gases on commencing to dig."

At Paris, in 1852, three men died from inhaling an escape of gas from coffins, and Mr. Fourcroy declared that all the grave-diggers he had examined showed signs of slow poisoning.

Mr. Chadwick affirms "that the sexton's vocation entailed a loss of one-third of the natural duration of life."

When the coffin of Francis I was opened, at the end of the last century, the dreadful vapors drove the men back, though he had lain there 250 years.

In 1845 the burial pits closed up at Louis during the "Black Death," in the fourteenth century, were opened, and the men employed were overpowered by the smell.

It is said that Marylebone grave-yard, closed for more than thirty years, smells, as does also St. John's Wood.

Dr. Selmi, of Mantua, has taken the pains to bottle the air of some cemeteries in calm weather. He finds it to contain an organic corpuscle, which he terms "septa-pneuma." This, administered to a pigeon, in solution, developed putrid fever and destroyed the bird on the third day. (See "Cremation of the Dead," by Dr. Pietra, Paris, p. 9.)

In our city the old burying-ground before referred to has been discontinued for public burials since 1864. Dr. Rauch, Secretary of State Board of Health, in a pamphlet, published in 1866, maintained that this cemetery contributed to the contamination of the water supply of the city.

During epidemics of yellow fever and cholera, the pernicious influence of intra-mural interments in this country has been most striking. The epidemic in New York in 1822 was considered to be the result of the overcrowded condition of Trinity church-yard cemetery before referred to.

Several instances are cited by Dr. Rauch, one of which was the epidemic at New Orleans in 1853. In the fourth district the mortality rose to 452 per thousand, which was more than double that of any other district in the city. In this district existed three extensive cemeteries, in which were buried the previous year more than three thousand bodies. In other districts the proximity to cemeteries seemed to aggravate the disease. Another instance was observed by Dr. Rauch personally during the epidemic of cholera in Burlington, Iowa, in 1850. No deaths occurred in the neighborhood of the city cemetery until about twenty interments had been made there, and then cases began to occur, and *always* in the direction from the cemetery in which the wind blew. Another instance was the epidemic at Norfolk and Portsmouth in 1855, reported by Dr. Bryant. Here 45 per cent. of the population died. Nearly all interments were made in the city, where the water-level is only six feet below the surface. The average depth of graves was about four feet, and, in many of them, three bodies were placed, one upon the other. These cemeteries were considered by Dr. Bryant a fruitful source of the disease.

In 1843, Edwin Chadwick presented to the Home Department a "Report on the Results of Special Inquiry into the Practice of Interment in Towns in the Kingdom of Great Britain and Ireland. Chadwick deduces, from the sum of evidence upon the subject, the conclusion: "That inasmuch as there appear to be no cases in which the emanations from human remains in an advanced stage of decomposition are not of a deleterious nature, so there is no case in which the liability to danger should be incurred, either by interment or by entombment in vaults, which is the most dangerous amidst the dwellings of the living; it being established, as a general conclusion, in respect to the physical circumstances of interment, from which *no adequate grounds of exception have been established*, that all interments in towns, where bodies decompose, contribute to the mass of atmospheric impurity which is injurious to the public health."

[TO BE CONTINUED.]

